# Rethinking the Management of Optic Pathway Gliomas: A Single Center Experience

**DOI:** 10.3389/fsurg.2022.890875

**Published:** 2022-06-16

**Authors:** Giada Del Baldo, Antonella Cacchione, Vito Andrea Dell’Anna, Pietro Merli, Giovanna Stefania Colafati, Antonio Marrazzo, Sabrina Rossi, Isabella Giovannoni, Sabina Barresi, Annalisa Deodati, Paola Valente, Elisabetta Ferretti, Mara Capece, Angela Mastronuzzi, Andrea Carai

**Affiliations:** ^1^ Department of Pediatric Haematology and Oncology, and Cell and Gene Therapy, Bambino Gesù Children’s Hospital, IRCCS, Rome, Italy; ^2^ Oncological Neuroradiology Unit, Imaging Department, Bambino Gesù Children’s Hospital, IRCCS, Rome, Italy; ^3^ Radiology and Neuro-radiology Unit, Ospedale Santissima Annunziata, Taranto, Italy; ^4^ Pathology Unit, Department of Laboratories, Bambino Gesù Children’s Hospital, IRCCS, Rome, Italy; ^5^ University Pediatric Hospital Department, Bambino Gesù Children’s Hospital, IRCCS, Rome, Italy; ^6^ Ophthalmology Department, Bambino Gesu’ Children’s Hospital, IRCCS, Rome, Italy; ^7^ Department of Experimental Medicine, Sapienza University, Rome, Italy; ^8^ Department of Neurosurgery, Università Politecnica delle Marche, Ancona, Italy; ^9^ Department of Neurosciences, Neurosurgery Unit, Bambino Gesù Children’s Hospital, IRCCS, Rome, Italy

**Keywords:** optic pathway gliomas, optic pathway surgery, BRAF, molecular target, pediatric neuro-oncology

## Abstract

**Background:**

Optic pathway gliomas (OPGs) are rare neoplasms in children with an unpredictable clinical course. Approximately 15% of OPGs occur in patients affected by neurofibromatosis type 1 (NF1): the clinical course of these cases is more indolently than sporadic ones, and NF1 patients less frequently require treatment including surgery. Instead, over 90% of sporadic OPGs require one or more therapeutic approaches. The management of OPG is controversial. They are also characterized by a high risk of morbidity including hypothalamic damage, endocrine deficits, visual deficit and/or neurological impairment.

**Materials and Methods:**

In this paper, we evaluated visual and endocrinological outcomes of a population of OPG followed at our center from 2013 to 2021, with a particular emphasis on the role of surgery.

**Results:**

Twenty-six patients were included in this study (mean age of 40.7 months). Tumor location on imaging was described by the Dodge classification. Five cases had NF 1. Thirteen cases received biopsy and 13 were partially resected. Histopathology revealed 19 cases of pilocytic astrocytomas, 2 pilomyxoid astrocytoma and 5 ganglioglioma. All the patients required a post-surgical adjuvant treatment according to current indications for low-grade gliomas. Molecular studies (BRAF status and mTOR/pmTOR pathway) have been performed in 24/26 patients, following for the use of target therapy in 11 of these patients. In our study we found that patients underwent biopsy have a better visual and endocrinological outcomes rather than patients with a tumor debulking. The five-year overall survival rate is 98% with a mean follow-up of 60 months.

**Conclusions:**

Many children with OPGs survive with a residual tumor. They suffer from chronic diseases such as endocrine dysfunction, visual disturbance, motor deficits and poor quality of life. All patients need comprehensive diagnostic work-up including neuroimaging, clinical evaluations and neuropathology approach; at the same time, they need therapeutic decisions and concepts for the choice of timing and type of neurosurgical intervention, chemotherapy and target therapy as well as surveillance and rehabilitation to maximize survival and overall functional outcomes. Our study showed that minimal invasive surgery with the purpose of molecular characterization of the tumor is desirable to reduce morbidity correlate to surgery.

## Introduction

Optic pathway gliomas (OPGs) account for 2%–7% of all pediatric central nervous system (CNS) tumors (1, 2). OPGs are typically associated to neurofibromatosis type 1 (NF1), can nonetheless develop sporadically in 20%–30% of the cases (3). OPGs are low-grade gliomas (LGG) potentially involving any portion of the optic pathways, with variable extension to the hypothalamus (4–6).

Their unpredictable natural course, including the heterogeneous presentation, possible spontaneous regression, and poor correlation between magnetic resonance imaging features (MRI) and visual acuity (VA) outcomes, renders management and prognosis usually challenging (7).

Heterogeneity is caused by different factors such as: age at presentation, NF1 status, tumor biology and disease location (8–10). Generally, NF1-associated OPGs are infiltrative, involving the optic nerves, chiasm, and/or posterior optic pathways and rarely require treatment (6, 11–14). On the other hand, sporadic OPGs frequently present earlier in life with larger tumors often arising from the chiasm, have a higher risk of progression (11–13) and require therapeutic intervention in the 90% of cases (15, 16).

MRI with dedicated sequences is indicated to confirm diagnosis and allow disease staging.

The classical Dodge staging system for OPGs classifies tumors based on anatomy with involvement of either the optic nerves, the optic chiasm, and the hypothalamus with associated structures (17). More recently, a modified Dodge classification has been developed, allowing a more detailed description of tumor involvement at multiple anatomical locations along the visual pathways (18).

Although being usually characterized by an indolent and favorable course, with over 90% 5-year overall survival (OS) rates, children with OPGs can experience significant chronic visual, neurological and endocrinological impairment (19, 20). Without reliable clinical indicators to predict the development of OPGs, careful surveillance for early signs of vision loss is essential (21).

The role of surgery has traditionally been very limited, due to tumor infiltration of eloquent brain structures and functional impairment associated with resection (22, 23).

Standard chemotherapy regimens include combinations of carboplatin and vincristine or vinblastine monotherapy (24, 25). More recently, molecular profiling of OPG has shown recurrent molecular lesions, such as MAPK pathway activation, opening the way to targeted approaches for the treatment of these children.

The purpose of our study was to retrospectively review our mono-institutional cohort of children with OPG, to determine the impact of surgery to support decision-making in the management of this challenging disease.

## Materials and Methods

We retrospectively examined patients referred for OPG to Bambino Gesù Children’s Hospital in Rome who underwent surgery from 2013 to 2021.

Inclusion criteria included: radiological diagnosis of OPG, age range 0–18 years, surgical procedure.

Collected variables included: age, gender, clinical presentation, NF1 status, Dodge stage, surgical strategy, histological and molecular features, visual and endocrinological assessment at diagnosis and after 3 months from surgery, treatment modalities and outcome.

The study was approved by the Institutional Review Board (IRB) and was conducted in accordance with the Helsinki Declaration. Written informed consent was obtained from all the patients or legal guardians.

### Radiological Assessment

Radiological images were reviewed at diagnosis, post-surgery, every six months during any treatment and at last follow-up for each patient.

Three Tesla MRI (Siemens Magnetom Skyra, Erlangen, Germany) studies were acquired using a standardized pediatric protocol including axial and coronal T2-weighted sequences, axial FLAIR, diffusion-weighted imaging (DWI) and susceptibility weighted imaging (SWI), pre- and post-contrast axial and volumetric T1- weighted sequences. In all patients, a spinal cord evaluation was also performed with post-contrast axial and sagittal T1- weighted sequences, and axial and sagittal T2-weighted sequences. Non-cooperating children underwent to general anesthesia. We adopted the Dodge classification for tumor location (17): optic nerve alone (stage 1), optic chiasm with/without optic nerve (stage 2), and post-chiasm with or without hypothalamus involvement (stage 3).

### Surgery

All procedures were performed by a dedicated pediatric neurosurgical team. Indication for surgery was discussed during a multidisciplinary tumor board in all cases. Purpose of surgery included both biopsy and debulking of lesions to alleviate symptomatic mass effect. Neurosurgical technique was adapted to lesion site and resection objective.

Lesions involving optic nerves and chiasm were approached by pterional craniotomy and microsurgical technique. Whenever significant extension to the third ventricle was present available options included endoscopic robot assisted procedures or microsurgical approaches including anterior transcallosal or frontal transcortical access to the lateral ventricle followed by transforaminal approach to the third ventricle. For lesions extending to the deep white matter, robot assisted stereotactic biopsy and tailors microsurgical approaches were considered.

All procedures were performed with navigation assistance and intraoperative monitoring and mapping as indicated to maximize preservation of eloquent areas.

### Histopathological and Molecular Characterization

Tumor sample was firstly used for histopathologic diagnosis. Available tumors tissue samples were centrally reviewed by expert neuropathologists. Immunohistochemistry (IHC) was carried out on formalin-fixed paraffin-embedded sections using an automated immunostainer (Dako Omnis).

Since 2018, all samples were investigated for BRAFv600E status and/or KIAA1549-BRAF fusion. Moreover, immunostaining for mTOR and p-mTOR was carried out. Previous samples were retrospectively analyzed for the same molecular aberrations.

Primary antibodies directed mTOR (clone 7C10 1:50, high pH, Cell Signaling Technology) and p-mTORSer2448 (D9C2, 1:100, high pH, Cell Signaling Technology) were used.

DNA isolation from tumor tissue was performed using the NucleoSpin Tissue Kit (Macherey-Nagel, Düren, Germany), according to the manufacturer’s instructions. Detection of the main mutations of codon 600 of the gene BRAF was performed using EasyPGX® ready BRAF (Diatech Pharmacogenetics). 10–25 ng of DNA per mix were used allowing the coamplification of one or more mutated alleles plus an endogenous control gene.

After obtaining informed consent for the genetic analyses, NGS panel was performed on total RNA extracted from formalin-fixed and paraffin-embedded sections (FFPE) of the tumor using ReliaPrep™ FFPE Total RNA kit (Promega, Wisconsin, USA). The quantity of RNA extracted was measured using Qubit fluorometric quantification system (Agilent). Two hundred nanogrammes of RNA were used for library preparation with the Archer® Universal RNA Reagent Kit for Illumina®, Archer MBC adapters, and our custom designed Gene Specific Primer (GSP) Pool kit. The sequencing run was performed using the Illumina MiSeQ platform. NGS data were analyzed using Archer Data Analysis Software v6.2.3.

RNA was extracted from formalin-fixed paraffin-embedded tumor tissue following ReliaPrep™ FFPE Total RNA kit (Promega) and quality and quantity of RNA samples was ascertained with the use of Agilent 2,200 Tapestation system (Agilent Technologies). Mean RNA integrity number (RIN) was 5.2 (range 3.0–6.9).

The SureSelect XT HS2 RNA kit (Agilent Technologies) was used to prepare RNA sequencing libraries from 300 ng of total RNA according to the manufacturer’s protocol. Libraries were pooled and sequencing run was performed in paired-end mode using the NextSeQ 550 system (Illumina, San Diego, California) generating at least 30 million reads per sample. Raw reads were aligned to the reference human genome (UCSC-Build38) using STAR (2.5.3a) algorithm.

Candidate fusion transcripts were identified by means of RNACocktail (V.0.3) using the FusionCatcher tool with default parameters. Only candidate fusions with (1) at least two unique reads (i.e. unique mapping positions) spanning the fusion breakpoint, and (2) not found in healthy control populations were considered reliable.

### Ophthalmologic Assessment

Visual performance data were collected at the time of diagnosis and six months after surgery.

Assessments included VA and Optical Coherence Tomography (OCT); visual field testing was excluded from this analysis for the well described bias depending on patient’s cooperation.

VA was measured using Teller Acuity Card grating acuity (Stereo Optical, Chicago, Illinois, USA) in children younger than 2.5 years old.

In older patients, VA was assessed using Snellen charts converted to the nomenclature using logarithm of the minimal angle of resolution (log MAR). A Snellen score of 20/20 converts to a logMAR of 0.0, 20/40 to a logMAR of 0.30, 20/100 to a logMAR of 0.69.

OCT is a non-invasive objective imaging modality that allows for a precise measurement of retinal nerve fiber layer (RNFL) thickness in OPGs. The best balanced cut off values of each analyzed sector have been referred to the RNFL thickness ranges proposed by Parrozzani et al. (26) and evaluation have been clustered as stable or worsened, with the cut off RNFL thickness reduction greater than 10% (in one or more quadrants or global average).

### Endocrinological Assessment

Endocrinological data were collected at the time of diagnosis and six months after surgery.

Clinical and auxological data, IGF-I, IGFBP-3, FT4, TSH, FSH, LH, testosterone, 17-beta-estradiol, ACTH, cortisol, glucose and electrolytes serum levels were retrospectively collected. Hormonal detection was measured by chemiluminescent immunometric assay (Immulite 2000 XPi, Siemens). GH hypersecretion (GHH) was defined as either IGF-1 and IGFBP-3 levels >97th percentileb (27, 28). GH deficiency (GHD) was defined as reduction height velocity over a period of 6 months and a GH peak less than10 ng/mL on 2 different provocative tests (arginine, clonidine or insulin tolerance test). Central precocious puberty (CPP) was defined as the appearance of secondary sexual characteristic before 8 years of age for girls and before 9 years of age for boys, and altered Gn-RH test. Thyrotropin deficiency was defined as low TSH levels with inappropriately low or normal FT4 levels based on age range values. ACTH deficiency was defined on cortisol peak reduction (≤500 mmol/L) during insulin tolerance test or ACTH test.

### Treatment

Chemotherapy was administered according to SIOP LGG 2004 indications. Based on molecular profile, targeted treatments included everolimus, trametinib, selumetinib and vemurafenib.

### Statistical Analysis

Data entry and cleaning were performed in Microsoft Excel. Data analyses were performed using GraphPad Prism software 9.0.

Type of surgery (biopsy or microsurgical debulking) was correlated with visual and endocrinological outcome for the whole population.

For endocrinological outcome patients were stratified in 3 groups: no hormonal deficits, one endocrinological deficit and two or more deficits. Concerning ophthalmological outcomes, VA was clustered into three groups:<0.2 logMAR (normal vision), 0.02–0.6 logMAR (mild impairment of vision), logMAR > 0.7 (severe visual impairment) and for OCT evaluation RNFL thickness reduction was classified in two groups: (1) greater than 10% (in one or more quadrants or global average (2) less than 10%.

Endocrinological and visual outcome were compared before and after surgery. Comparison tests included Fisher’s exact test and chi-squared test, when appropriate.

All comparison tests were two-sided and considered significant at the 5% level.

## Results

We retrospectively identified 26 consecutive patients fulfilling inclusion criteria. Median age at diagnosis was 40.7 months (range 3.0–201.7). There were 13 males and 13 females. Five patients (19%) had a clinical and/or genetic diagnosis of NF1. Thirteen cases (50%) were diagnosed for evidence of visual symptoms including decreased VA and nystagmus. Two patients (8%) were diagnosed during a screening scan for NF1. Six children (23%) presented for headache and vomiting. Five patients (19%) were diagnosed after detection of CPP. Clinical features of the study population are summarized in Table 1.

**Table 1:** Population characteristics.

OPGs	*n* = 26
Gender	
** **Male	13/26 (50%)
** **Female	13/26 (50%)
Age at diagnosis (months)	
** **Median (months)	40.7
** **Range (months)	3.0–201.7
Dodge classification	
** **Stage 1	1/26 (4%)
** **Stage 2	6/26 (23%)
** **Stage 3	19/26 (73%)
Hypothalamic involvement	18/26 (70%)
Type	
** **NF1	5/26 (19%)
** **Sporadic	21/26 (81%)
Surgery	
** **Biopsy	13/26 (50%)
** **Microsurgical debulking	13/26 (50%)
Ventriculoperitoneal shunt (VPS)	9/26 (35%)
Upfront Treatment	26/26 (100%)
** **Chemotherapy	23/26 (88%)
** **Target therapy	3/26 (12%)
Second-third line treatment	10/26 (42%)
** **Chemotherapy 2^nd^line	2/26 (8%)
** **Target therapy	8/26 (31%)

### Surgery

Biopsy was performed in 13 cases: robot-assisted stereotactic needle biopsy (23%), endoscopic transventricular biopsy (38.5%) and microsurgical biopsy (38.5%).

Microsurgical debulking was performed in the remaining half of the population.

Nine patients out of 26 required ventriculoperitoneal shunt (VPS) positioning for evidence of hydrocephalus. The rate of shunting was higher in patients who had undergone microsurgical debulking of OPG (6/9, 67%).

From 2013 to 2017, 5/15 patients underwent biopsy (33%); from 2018 to 2021 biopsy was performed on 7/11 patients (63%).

### Histopathological and Molecular Characterization

Histological features of OPG were available for the whole population: pilocytic astrocytoma in 19/26 (73%), ganglioglioma in 5/26 (19%) and pilomyxoid astrocytoma in 2/26 (8%). The BRAFv600E mutation was investigated in 20/26 (76%) and the mutation was found in 5/20 cases analyzed (25%). KIAA1549-BRAF fusion was investigated in 15/26 (57%) and resulted positive in 10/15 (67%). Evaluation of immunostainings for mTOR/p-mTOR pathway was performed in 23/26 (88%) and an overexpression was confirmed in 22/23 cases (96%).

An overview of surgical details and molecular findings is presented in Table 2.

**Table 2:** Surgical treatment and molecular findings.

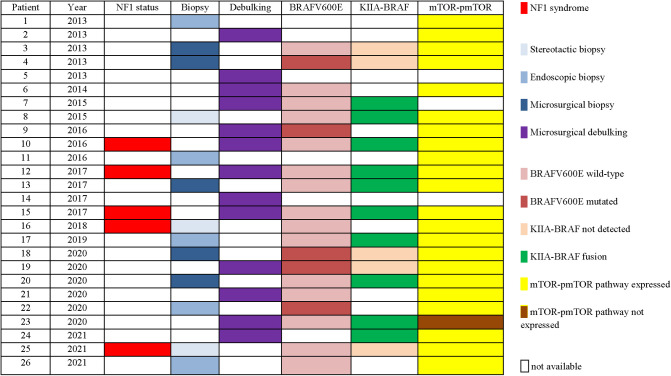

### Treatment

All 26 patients received a post-surgical adjuvant treatment: chemotherapy in 23/26 patients and target first line therapy in 3 cases diagnosed after 2018 and with mild visual impairment.

Ten patients treated with standard chemotherapy progressed at follow-up. Median time to progression for all patients was 38 months (range 11–108). Second line treatment for disease progression was carried out in 10/26 patients (2/10 chemotherapy and 8/10 target therapy).

### Ophthalmologic Assessment

Eleven children (42%) presented a pathological OCT (comparing with the most sensitive cut-off values of each RNFL analyzed in any optical nerve sector) before any surgical approach; the analysis after biopsy showed that 85% of the patients remained stable and 15% worsened (>10% reduction of the thickness). OCT worsened in 62% of the children after debulking surgery. Regarding VA, 9/26 patients (35%, *n* = 9) had normal/near normal vision (group 1, <0.2 LogMAR); 54% (*n* = 14) had mild/moderate impairment (group 2, 0.2 –0.6 LogMAR), and 11% (*n* = 3) had severe visual impairment (group 3, >0.7 LogMAR). VA evaluation after biopsy documented worsening of VA in 1 child (8%) vs 7/13 patients (54%) in the surgical debulking cohort (Table 3).

**Table 3:** VA and OCT variations after surgical procedure.

	*N* patients	Stable	Worse
VA^a^ post biopsy	13/26	12/13 (92%)	1/13 (8%)
VA post surgery	13/26	6/13 (46%)	7/13 (54%)
OCT^b^ post biopsy	13/26	11/13 (85%)	3/13 (15%)
OCT post surgery	13/26	5/13 (38%)	8/13 (62%)

^a^ *Change in VA defined between the three LogMAR clusters (>0.2; 0.2–0.6; >0.7)*.

^b^ *RNFL thickness reduction greater than 10% (in one or more quadrants) was evaluated as worsening OCT*.

Children undergoing biopsy were significantly less likely to have a VA outcome of >0.7 LogMAR (p 0.03) and a thickness reduction > 10% (p 0.04) compared with those undergoing debulking surgery (Table 4).

**Table 4:** Correlation of VA, OCT and hormonal deficits according to different surgical approach (biopsy vs debulking).

	Debulking	Biopsy	OR (95% CI)	*p*-value
Worsened VA	7/13 (54%)	1/13 (8%)	14 (1.4–168.0)	0.03
Worsened OCT	8/13 (62%)	2/13 (15%)	8.8 (1.3–48.0)	0.04
Worsened hormone deficits	8/13 (62%)	1/13 (8%)	19.2 (1.9–226.6)	0.01

### Endocrinological Assessment

Endocrine dysfunctions were identified in 11 patients (42%) before any surgical approach.

Globally, nine out of 26 patients (35%) presented a worsening endocrinological outcome after surgery; 8/13 patients (62%) after debulking and 1/13 after biopsy (8%).

Following surgery, 7 out of 15 patients without hormonal defects at diagnosis (46%) presented a new hormonal deficit: in one case after biopsy (14%) and in six (86%) after microsurgical debulking. In 2 cases out of 11 patients with endocrinological defects at diagnosis (18%), the pre-existing hormonal defects worsened after surgery, both having undergone debulking. In 9/11 cases (81%) the hormonal deficits remained stable (7/9 cases after biopsy and 2/9 after microsurgical debulking). In 7 cases (27%) no hormonal deficit was detected at follow-up.

As showed in Table 4, children treated with a bioptic approach were significantly less likely to have endocrinological impairment in comparison to children treated with microsurgical debulking (p 0.01).

Endocrinological defects before and after surgery are summarized in Table 5.

**Table 5:** Endocrinological defects before and after surgery.

	Microsurgical debulking	Biopsy
Hormonal deficits at diagnosis	4/13 (31%)	7/13 (54%)
Stable hormonal status	2/4 (50%)	7/7 (100%)
Worse hormonal status	2/4 (50%)	0/7 (0)
New hormonal deficits after surgery	6/9 (67%)	1/6 (16%)

Notably, only 3 patients presented worsening on all the three parameters (VA, OCT and hormone deficits) and all of them had undergone a microsurgical debulking procedure and needed a VPS implant.

Median OS at last follow-up for patients in this study was 65.5 months (range 6.6–156.8). Only one patient died due to severe neurological impairments from tumor growth 61 months after diagnosis.

## Discussion

OPGs represent 2%–7% of all pediatric intracranial tumors with a peak of incidence in children younger than 5 years (29–31) and affected by NF1. In contrast to the good prognosis *quoad vitam*, these tumors are associated with significant potential morbidity, the most important of which concern visual impairment and endocrinological disfunction (2, 9, 32).

Their management is still controversial ranging from conservative approach to multimodality treatments including surgery, chemotherapy and radiotherapy in selected cases (22, 30, 31, 33). The decision to treat and the choice of the type and time of treatment depends on different clinical and radiological aspects but also on the expertise of the treating team and available resources.

The role of surgical resection has recently been revised, on the bases of previously reported unacceptable morbidity, and limited to bioptic procedures in selected cases and microsurgical debulking to alleviate mass effect symptoms (23, 34). Indications for surgery are even stricter in NF1 patients, in which MR is pathognomonic of OPG and histological confirmation is not mandatory to prompt treatment.

However, the ever-growing neurosurgical technical armamentarium opens new possibilities to push boundaries and reconsider a role for surgery in the management of these children.

Nonetheless, many aspects remain to be elucidated in respect to the potential benefits of high-risk surgical resections.

Surgical biopsy has often been of limited utility, the vast majority of these lesions being LGG susceptible to the same oncological treatment, even in non-NF1 children.

A greater understanding of the genetic landscape of LGG and the development of novel drugs that target some of these molecular aberrations have heralded a new focus upon molecularly driven treatments, with the goal to minimize toxicity and maximize survival and functional outcomes (35).

It is now well understood that abnormal MAPK pathway activation is the most frequent genetic aberration detected in pediatric LGG, most commonly resulting from activation of the BRAF oncogene (35–37). The two most common aberrations described are an activating point mutation BRAFv600E and a tandem duplication resulting in a KIAA1549-BRAF fusion. This fusion results in a transcript in which the kinase domain of the BRAF gene is fused to a gene of unknown function (KIAA1549) (38), leading to increased BRAF activation of the downstream MEK signaling cascade. Moreover, other potential driver mutations or fusions in sporadic OPGs include the KRAS, FGFR1, PTPN11, RAF1, and NTRK2 genes (39–43).

Clinical trials of oral targeted MAPK inhibitors (trametinib, dabrafenib and selumetinib) have shown efficacy in pediatric LGG (44), and upfront studies of these novel therapies are ongoing (45). Trametinib, a reversible and highly selective inhibitor of MEK1/MEK2 activation and kinase activity, has also shown to be effective, alone or in combination with dabrafenib, in phase I studies (38, 39) and case series reports (40, 41, 46). A phase II study to confirm the efficacy and safety of trametinib as single agent in NF1-associated LGG and non-NF1 gliomas with either KIAA1549-BRAF fusion or activation of the MAPK/ERK pathway, is ongoing (NCT03363217). Vemurafenib, a competitive and selective inhibitor of the BRAFV600E mutant ATP-binding domain, has proved well-tolerated and active as single agent in a case series of pediatric patients with BRAFv600E-positive LGG (47). Furthermore, as mTOR pathway activation in LGGs is well known, orally administration of everolimus has been studied and demonstrated disease stability also as upfront line (48).

The choice of the specific molecular inhibitor depends on the underlying biology, making biopsy essential (22, 23) to collect biological samples to perform molecular investigations to identify the best tailored treatment (49).

In our series, bioptic procedures were sufficient to allow molecular characterization of tumors in almost all cases, with an acceptable functional outcome.

In fact, both visual performance and endocrinological function were spared in the majority of cases when surgery was limited to biopsy independently from the technique used.

Visual deterioration after surgery was found to be higher after debulking (VA decline 54% and OCT worsening in 62%) than after biopsy (VA decline 8%, OCT worsening 15%).

Similarly, endocrinological worsening was found after surgery in 9/26 cases (35%), 8 of which had undergone microsurgical debulking. The difference in endocrinological impairment after surgery between biopsied (8%) and resected (62%) OPG was statistically significant. This evidence supports the notion that the incidence of hypopituitarism is higher with extensive resection.

Interestingly, we documented a change in the trend of surgical indications with an increased number of biopsies (63%) in the last 3 years compared to earlier ones (33%).

Although partial resection is an option in optic pathway glioma, it does not confer an advantage in the chances of disease control. In recent years our policy was to minimize surgical manipulation and functional complications, for the sake of faster recovery from surgery and improved quality of life.

Moreover, we found a high rate of targetable lesions for which effective and safe oral drugs were available. Considering that OPGs morbidity has a greater impact than mortality, this aspect becomes of paramount importance.

In pediatric OPG cases, in order to carry the minor possible damage to visual and endocrinological functions, it seems important to determine the treatment policy with a long-term perspective. We advocate the collection of tumor tissue to identify the molecular landscape and consequently use a target therapy approach in this population.

Our study has several limitations, including small and retrospective sample from a single institution. Moreover, sharing of surgical strategy in the setting of a multidisciplinary tumor board and development of a dedicated surgical oncology team must be accounted before translating results to different settings. However, we believe that our observations might deserve further studies in larger populations to allow generalization of our promising results.

## Conclusions

The treatment approach of pediatric OPGs is still controversial. The purpose of management must be to reduce long-term disability.

Preliminary reports of efficacy and safety of molecularly driven targeted therapies prompts a reconsideration of the management strategy of these difficult cases.

In this scenario, a strong multidisciplinary approach might justify a growing role for surgery. Our preliminary data suggest the possibility of tissue sampling in virtually all cases with acceptable morbidity compared to more extensive surgical approaches.

## Data Availability

The original contributions presented in the study are included in the article/supplementary material, further inquiries can be directed to the corresponding author/s.
